# Nerve injury in severe trauma with upper extremity involvement: evaluation of 49,382 patients from the TraumaRegister DGU® between 2002 and 2015

**DOI:** 10.1186/s13049-018-0546-6

**Published:** 2018-09-10

**Authors:** Torge Huckhagel, Jakob Nüchtern, Jan Regelsberger, Rolf Lefering, TraumaRegister DGU

**Affiliations:** 1 Department of Neurosurgery, University Medical Center Hamburg- Eppendorf, Hamburg, Germany; 2 Department of Trauma, Hand and Reconstructive Surgery, University Medical Center Hamburg-Eppendorf, Hamburg, Germany; 3 Institute for Research in Operative Medicine (IFOM), University of Witten / Herdecke, Cologne, Germany; 4 Committee on Emergency Medicine, Intensive Care and Trauma Management (Sektion NIS) of the German Trauma Society (DGU), Berlin, Germany

## Abstract

**Background:**

Peripheral nerve injury (PNI) as an adjunct lesion in patients with upper extremity trauma has not been investigated in a Central European setting so far, despite of its devastating long-term consequences. This study evaluates a large multinational trauma registry for prevalence, mechanisms, injury severity and outcome characteristics of upper limb nerve lesions.

**Methods:**

After formal approval the TraumaRegister DGU® (TR-DGU) was searched for severely injured cases with upper extremity involvement between 2002 and 2015. Patients were separated into two cohorts with regard to presence of an accompanying nerve injury. For all cases demographic data, trauma mechanism, concomitant lesions, severity of injury and outcome characteristics were obtained and group comparisons performed.

**Results:**

About 3,3% of all trauma patients with upper limb affection (n = 49,382) revealed additional nerve injuries. PNI cases were more likely of male gender (78,6% vs.73,2%) and tended to be significantly younger than their counterparts without nerve lesions (mean age 40,6 y vs. 47,2 y). Motorcycle accidents were the most frequently encountered single cause of injury in PNI patients (32,5%), whereas control cases primarily sustained their trauma from high or low falls (32,2%). Typical lesions recognized in PNI patients were fractures of the humerus (37,2%) or ulna (20,3%), vascular lacerations (arterial 10,9%; venous 2,4%) and extensive soft tissue damage (21,3%). Despite of similar average trauma severity in both groups patients with nerve affection had a longer primary hospital stay (30,6 d vs. 24,2 d) and required more subsequent inpatient rehabilitation (36,0% vs. 29,2%).

**Conclusion:**

PNI complicating upper extremity trauma might be more commonly encountered in Central Europe than suggested by previous foreign studies. PNI typically affect males of young age who show significantly increased length of hospitalization and subsequent need for inpatient rehabilitation. Hence these lesions induce extraordinary high financial expenses besides their impact on health related quality of life for the individual patient. Further research is necessary to develop specific prevention strategies for this kind of trauma.

## Background

Extremity involvement is very commonly seen in multiply injured patients. A single-center evaluation of injury patterns revealed extremity trauma including the pelvic girdle in 53% of 1599 consecutive shock room trauma patients [1]. Data from the TraumaRegister DGU® showed a significant number of extremity lesions (AIS>/=2) in 58,6% of 24,885 patients with an ISS >/= 16 [2]. The upper limbs generally seem to be affected in about 21,9% to 32,8% of trauma patients [2, 3]. There is a scarcity of studies within the medical literature which report on the frequency and context of additional nerve injury aggravating specifically upper extremity trauma. A comprehensive study of 5777 trauma victims between 1986 and 1996 performed by Noble and colleagues identified 162 patients with upper or lower extremity peripheral nerve injuries (PNI) which would be a fraction of 2,8% of all trauma patients [4]. A retrospective analysis of the MarketScan Commercial Claims and Encounters Database (The MEDSTAT Group) refers a 1,64% incidence of nerve injuries within 90 days after upper or lower limb trauma [5]. The aforementioned studies have been performed within the United States and Canada. Other series from Mexico and Iran report PNI prevalences of 1,1-1,3% [6, 7]. Trauma prevalence, epidemiological features, injury mechanisms and patterns may vary due to different socioeconomic factors and law regulations. Hence data from overseas should not be transferred inconsiderately into the European context. Given the high rate of neuropathic pain, functional deficits and overall reduced quality of life following traumatic neuropathy there is a general need for a profound assessment of extremity PNI to provide health practicioners and officials with essential information for patient care and resource allocation [8]. A multinational registry based evaluation of nerve injuries in lower extremity trauma patients has already been published previously [9]. Thus, this survey aims at scrutinising the same comprehensive trauma registry to get detailed insight into prevalence rate of peripheral nerve involvement complicating upper limb trauma in Europe. Furthermore we attempt to identify specific trauma patterns which put patients at particular risk for additional upper extremity nerve injury by contrasting them to trauma cases without PNI. Additionally we provide in-depth information on demographic data, accident cause, injury severity, treatment and outcome characteristics of severe trauma patients with concomitant upper limb PNI.

## Methods

**Fig. 1:**
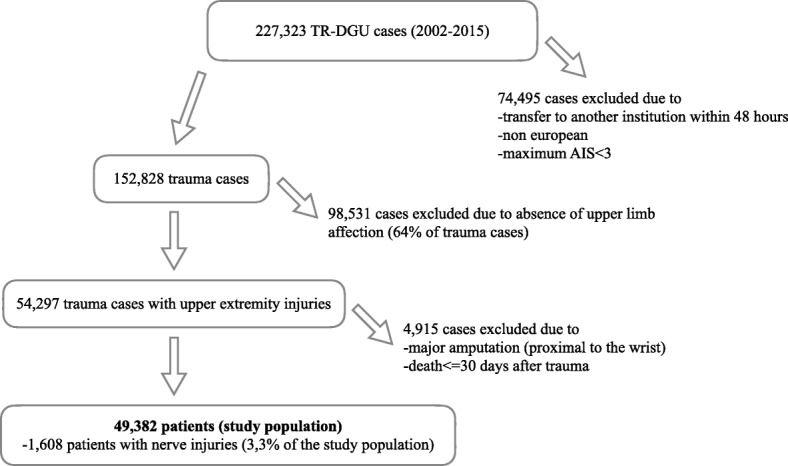
flowchart study population_ upper limb nerve injury

All data presented within this study were queried from the TraumaRegister DGU® (TR-DGU). The TR-DGU of the German Trauma Society (Deutsche Gesellschaft für Unfallchirurgie, DGU) was founded in 1993. The aim of this multi-centre database is a pseudonymised and standardised documentation of severely injured patients. Data are collected prospectively in four consecutive time phases from the site of the accident until discharge from hospital: A) Pre-hospital phase, B) Emergency room and initial surgery, C) Intensive care unit and D) Discharge. The documentation includes detailed information on demographics, injury pattern, comorbidities, pre- and in-hospital management, course on intensive care unit, relevant laboratory findings including data on transfusion and outcome of each individual. The inclusion criterion is admission to hospital via emergency room with subsequent ICU/IMC surveillance or reach the hospital with vital signs and die before admission to ICU. The infrastructure for documentation, data management, and data analysis is provided by AUC - Academy for Trauma Surgery (AUC - Akademie der Unfallchirurgie GmbH), a company affiliated to the German Trauma Society. The scientific leadership is provided by the Committee on Emergency Medicine, Intensive Care and Trauma Management (Sektion NIS) of the German Trauma Society. The participating hospitals submit their pseudonymised data into a central database via a web-based application. Scientific data analysis is approved according to a peer review procedure established by Sektion NIS. The participating hospitals are primarily located in Germany (90%), but a rising number of hospitals of other countries contribute data as well (at the moment from Austria, Belgium, China, Finland, Luxembourg, Slovenia, Switzerland, The Netherlands, and the United Arab Emirates). A comprehensive list of all contributing institutions is available at the TraumaRegister DGU® website (www.traumaregister-dgu.de). Currently, approx. 33,000 cases from more than 600 hospitals are entered into the database per year. Participation in TR-DGU is voluntary. For hospitals associated with TraumaNetzwerk DGU® however, the entry of at least a basic data set is obligatory for reasons of quality assurance. The present study is in line with the publication guidelines of the TraumaRegister DGU® and registered as TR-DGU project ID 2017–002. The manuscript passed a formal TR-DGU review process and authorization from the review board has been achieved subsequently. We recognized more than 227,000 TR-DGU cases which were entered into the database between 2002 and 2015. We included only those patients from European hospitals who presented with at least one AIS of 3 or more in any body region to make sure that the investigated cohort only consists of seriously injured trauma victims. After exclusion of all cases without upper limb trauma the remaining 54,297 patients were screened for major amputations (by definition proximal to the wrist) and death earlier than 30 days after the initial incident which led to hospital admission. Patients fulfilling at least one of these characteristics were also excepted, because both instances derogate correct nerve injury evaluation. The remaining total study population comprises 49,382 patients. 87,5% of the investigated cases were primarily treated in German trauma centres. Flowchart (Fig. 1) demonstrates the detailed selection process applied for this study with underlying specifications. All cases were investigated regarding epidemiological data, trauma etiology and mechanism, damage patterns including further impairment of anatomical structures and body regions, injury severity, outcome measures and nerve specific surgical interventions. Those patients with nerve lesions were compared to the upper limb trauma cohort without associated peripheral neurological deficit. Additionally we utilized the recently established TR-DGU cost estimator to calculate mean treatment expenditures for both groups [10]. Meeting the TR-DGU coding principles represented by the reduced version of the AIS 2005 nerve trauma of the upper limb (arm and hand) can be categorized as minor lesion (AIS 2005 code: 730699.1) or major injury (AIS 2005 code: 730604.2). Data retrieved from TraumaRegister DGU® are presented in a descriptive mode as percentages and frequencies. Where suitably tables also include 95% confidence intervals (CI) and standard deviations (SD) for these ratios. Additionally central tendency measures (mean and median) are given, if appropriate.

## Results

### Epidemiology

1608 of all included patients with upper limb injury (*n* = 49,382) exhibited adjunctive nerve involvement (3,3%). These trauma victims (PNI group) were more likely of male sex compared to the control group without additional nerve lesions (78,6% PNI group versus 73,2% control group). Mean (median) age of patients with concomitant upper extremity nerve lesion was more than 6 ys (8 ys) lower compared to their counterparts. Especially the age group which comprises the main part of the workforce (between 15 and 59 ys) is more commonly represented in PNI patients (82,7% PNI group versus 68,9% control group). Detailed epidemiological information including subdivision into 5 distinct age bands is displayed in Table 1.

**Table 1:** Epidemiology_ upper limb nerve injury

	control group	PNI group
proportion of all patients (%)	96,7 (CI 95,9 - 97,6)	3,3 (CI 3,1 - 3,4)
male (%)	73,2 (CI 72,5 - 74,0)	78,6 (CI 74,3–83,1)
mean / median age (years)	47,2 / 47,0 (SD 20,2)	40,6 / 39,0 (SD 17,6)
1–15 years (%)	2,8 (CI 2,7 - 3,0)	1,7 (CI 1,1 - 2,5)
16–59 years (%)	68,9 (CI 68,1–69,6)	82,7 (CI 78,3–87,3)
60–69 years (%)	11,7 (CI 11,4–12,0)	8,6 (CI 7,2 - 10,1)
70–79 years (%)	10,3 (CI 10,0 - 10,6)	4,7 (CI 3,7 - 5,9)
80 years or more (%)	6,4 (CI 6,1 - 6,6)	2,3 (CI 1,6 - 3,2)

This table shows epidemiological data of 49,382 patients with upper extremity trauma (= all patients). Additionally to the mean and median age a further subdivision into 5 age bands is provided. *CI* Confidence Interval of 95%, *SD* Standard Deviation

### Trauma etiology and mechanisms

In both cohorts the majority of trauma cases was traffic related, but further subgroup division revealed different causative patterns. PNI occured mainly in association with motorbike (32,5%) and car collisions (26,9%), whereas high or low falls taken together (32,2%) and car crashes (25,4%) were the most commonly seen reasons for trauma in the control group. Interestingly bicycle accidents happened more frequently in the control group (9,5% control group versus 4,1% PNI group). With respect to the underlying mechanism the bulk of injuries in both collectives emerged in the context of blunt trauma (85,3% PNI group; 96,9% control group), but a remarkable proportion of PNI was associated also with penetrating incidents (14,7%). Comprehensive data is provided by Table 2.

**Table 2:** Etiology of trauma_ upper limb nerve injury

	control group	PNI group
car (%)	25,4 (CI 25,0 - 25,9)	26,9 (CI 24,3 - 29,7)
motorbike (%)	21,0 (CI 20,5 - 21,4)	32,5 (CI 29,6–35,5)
bicycle (%)	9,5 (CI 9,2 - 9,8)	4,1 (CI 3,1 - 5,2)
pedestrian (%)	7,0 (CI 6,8 - 7,3)	5,4 (CI 4,3 - 6,7)
fall > 3 m (%)	19,6 (CI 19,2 - 20,0)	13,5 (CI 11,7 - 15,5)
fall < 3 m (%)	12,6 (CI 12,3 - 12,9)	5,4 (CI 4,2 - 6,7)
other (%)	4,9 (CI 4,7 - 5,2)	12,3 (CI 10,6–14,3)
blunt (%)	96,9 (CI 96,0 - 97,8)	85,3 (CI 80,8 - 90,0)
penetrating (%)	3,1 (CI 2,9 - 3,3)	14,7 (CI 12,9 - 16,7)

Comparison of PNI and control group with regard to different causes and mechanisms of trauma. *CI* Confidence Interval of 95%

### Injury patterns

A juxtaposition of PNI and control group concerning significant traumatic involvement of other anatomic body regions (defined as AIS > 1) revealed marked distinctions for head, abdomen and lower extremities, whereas thorax, spine and pelvis were similarly often injured in both cohorts. Head trauma occured more frequently in non-PNI cases (43,9% control group versus 31,5% PNI group), while PNI patients showed more abdominal (25,1% PNI group versus 19,6% control group) and leg injuries (41,9% PNI group versus 37,1% control group). Almost equal distributions were encountered for additional thorax, spine and pelvic trauma in both samples. 66,4% of the PNI group and 65,5% of the control group had additional thorax lesions. Spine injuries were seen in 34,1% of PNI patients and 31,7% of the control goup (pelvic trauma: 20,7% PNI group versus 20,5% control group). Moreover PNI and control group were investigated for accompanying bone fractures and joint injuries of the upper limb. Shoulder lesions were more frequently observed in the control group (scapula 18,4%; clavicula 25,3%) compared to PNI patients (scapula 13,3%; clavicula 13,4%), but upper arm and ulnar forearm fractures were predominantly associated with PNI (humerus 37,2%; ulna 20,3%) contrasted to those patients belonging to the control group (humerus 15,7%; ulna 12,9%). The rate of radial fractures was only slightly different in both collectives (28,5% PNI group versus 25,1% control group). Also fractures of the carpals, metacarpal bones and fingers taken together occured almost equally frequent (12,9% PNI group; 12,0% control group). Both study populations showed very similar propensities regarding joint injuries of the upper extremity. Basically proximal joint dislocations were more commonly seen than their distally located counterparts (shoulder 6,7%; elbow 3,1%; wrist 1,6% of all cases). Vascular damage was significantly more often encountered in PNI compared to control cases. Arterial (venous) lesions arose in 10,9% (2,4%) of patients with concomitant nerve injury and 0,5% (0,1%) of the control group. Extensive soft tissue affection was also more commonly seen in PNI than in control cases (21,3% PNI group versus 15,8% control group). Detailed information can be obtained from Table 3.

**Table 3:** Concomitant lesions_ upper limb nerve injury

	control group	PNI group
head (%)_AIS > 1	43,9 (CI 43,3 - 44,5)	31,5 (CI 28,9–34,4)
thorax (%)_AIS > 1	65,5 (CI 64,8 - 66,2)	66,4 (CI 62,4 - 70,5)
abdomen(%)_AIS > 1	19,6 (CI 19,2 - 20,0)	25,1 (CI 22,7 - 27,7)
spine (%)_AIS > 1	31,7 (CI 31,2 - 32,2)	34,1 (CI 31,3 - 37,1)
pelvis (%)_AIS > 1	20,5 (CI 20,0 - 20,9)	20,7 (CI 18,5 - 23,1)
legs (%)_AIS > 1	37,1 (CI 36,5–37,6)	41,9 (CI 38,8 - 45,2)
bone_scapula (%)	18,4 (CI 18,0 - 18,8)	13,3 (CI 11,6–15,2)
bone_clavicula (%)	25,3 (CI 24,9 - 25,8)	13,4 (CI 11,6–15,3)
bone_upper arm (%)	15,7 (CI 15,3 - 16,1)	37,2 (CI 34,3 - 40,3)
bone_radius (%)	25,1 (CI 24,6 - 25,5)	28,5 (CI 26,0–31,3)
bone_ulna (%)	12,9 (CI 12,6–13,2)	20,3 (CI 18,1–22,6)
bone_hand (%)	12,0 (CI 11,7 - 12,4)	12,9 (CI 11,2 - 14,8)
joint_shoulder (%)	6,7 (CI 6,5 - 6,9)	7,0 (CI 5,7 - 8,4)
joint_elbow (%)	3,0 (CI 2,9 - 3,2)	5,7 (CI 4,6 - 6,9)
joint_wrist (%)	1,6 (CI 1,5 - 1,7)	2,1 (CI 1,5 - 3,0)
vessel_artery_arm (%)	0,5 (CI 0,4 - 0,6)	10,9 (CI 9,3 - 12,6)
vessel_vein_arm (%)	0,1 (CI 0,1 - 0,1)	2,4 (CI 1,7 - 3,3)
soft tissue_arm (%)	15,8 (CI 15,4–16,2)	21,3 (CI 19,1–23,6)

Specification of accompanying involvement of other body regions, bones, joints, vessels and soft tissue in PNI and control patients. *AIS* Abbreviated Injury Scale, *CI* Confidence Interval of 95%

### Severity of injury and treatment

PNI and control cases were almost identical with regard to mean trauma severity (PNI group ISS 22,4; control group ISS 22,5). Patients with additional nerve lesions presented with higher shock prevalences defined as maximum systolic blood pressure of 90 mmHg both prehospitally (17,3% PNI group versus 11,3% control group) and later on in the emergency room (13,1% PNI group versus 8,6% control group) which is in congruence with a higher rate of carriage by rescue helicopter for this population (36,0% PNI group; 28,8% control group). Usually patients of either group had to be treated at the ICU after hospital admission primarily (93,1% PNI group; 89,9% control group) for comparable mean time periods (11,3 days PNI group; 10,2 days control group). Interestingly the mean intubation time was slightly shorter in PNI cases (8,2 days PNI group; 9,9 days control group) despite of longer ICU treatment duration. Even though both cohorts did not differ much in terms of overall trauma severity (ISS) and ICU period, the average length of total hospital stay was profoundly different in both populations (30,6 days PNI group; 24,2 days control group). Longer hospitalisation in case of accompanying PNI compared to upper limb trauma without nerve affection resulted in more than 5000 Euro higher calculated treatment costs per patient utilizing the cost estimator which was established for the TR-DGU recently [10]. Table 4 adresses injury severity and treatment variables in depth. Eventually the PNI group was dichotomized according to the extension of the nerve lesion to evaluate the frequency of nerve specific surgical procedures. Following the TR-DGU coding principles nerve contusions (lacerations) were defined as minor (major) injuries. Those patients with minor nerve damage (about 1/3 of the PNI) had less surgical interventions than their counterparts with major deficits (14,8% minor PNI versus 20,6% major PNI).

**Table 4:** Severity of injury and treatment_ upper limb nerve injury

	control group	PNI group
ISS (mean/median)	22,4 / 20,0 (SD 10,4)	22,5 / 20,0 (SD 10,5)
carriage by rescue helicopter (%)	28,8 (CI 28,2 - 29,3)	36,0 (CI 32,95–39,32)
shock_prehospital (%)	11,3 (CI 11,0 - 11,7)	17,3 (CI 15,1 - 19,7)
shock_emergency room (%)	8,6 (CI 8,3 - 8,9)	13,1 (CI 11,3 - 15,1)
ICU treatment (%)	89,9 (CI 89,0 - 90,7)	93,1 (CI 88,4 - 97,9)
mean/median ICU treatment duration (days)	10,2 / 5,0 (SD 12,8)	11,3 / 6,0 (SD 13,3)
mean/median intubation time (days)	9,9 / 5,0 (SD 12,2)	8,2 / 4,0 (SD 10,0)
mean/median hospital stay (days)	24,2 / 18,0 (SD 21,7)	30,6 / 25,0 (SD 25,1)
calculated treatment costs per patient in Euro (mean/median)	22,523 / 14,730	27,632 / 20,035

Characterization of PNI and control patients with regard to distinct trauma severity indicators, ICU and total hospital length of stay as well as calculated treatment expenditures per case (currency: Euro). *CI* Confidence Interval of 95%, *ICU* Intensive Care Unit, *ISS* Injury Severity Score, *SD* Standard Deviation

### Outcome

We evaluated both cohorts according to their functional status by utilizing the Glasgow Outcome Scale (GOS) at the point of hospital discharge. Marked differences between PNI and control group were recorded for the rates of the higher functional performance scores (GOS 4 and 5), but the proportions of trauma cases with lower grades (GOS 1–3) were nearly equivalent in both cohorts Table 5. PNI patients reached GOS 4 (GOS 5) in 39,9% (49,1%), whereas the control group revealed GOS values of 4 [5] in 28,4% (59,8%) which represents a significant shift to worse neurological condition in PNI compared to control group patients despite of almost equally distributed mean trauma severity scores in both cohorts. Consequently patients with concomitant nerve injuries were more commonly sent to rehabilitation centres for further treatment (36,0% versus 29,2%). 49,1% of PNI and 54,5% of control cases were directly sent home from the hospital they were primarily admitted to without further inpatient care in another health institution.

**Table 5:** Outcome_ upper limb nerve injury

	control group	PNI group
GOS 1 (death > 30 days after trauma) (%)	0,6 (CI 0,5 - 0,7)	0,1 (CI 0,0 - 0,5)
GOS 2 (%)	1,7 (CI 1,6 - 1,8)	0,8 (CI 0,4 - 1,4)
GOS 3 (%)	9,6 (CI 9,3 - 9,9)	10,1 (CI 8,6–11,8)
GOS 4 (%)	28,4 (CI 27,9 - 28,9)	39,9 (CI 36,8 - 43,2)
GOS 5 (%)	59,8 (CI 59,0 - 60,5)	49,1 (CI 45,7–52,7)
discharge_home (%)	54,5 (CI 53,8 - 55,1)	49,1 (CI 45,7–52,6)
discharge_rehabilitation (%)	29,2 (CI 28,7 - 29,7)	36,0 (CI 33,1–39,0)
discharge_other hospital (%)	12,4 (CI 12,1 - 12,8)	12,2 (CI 10,5–14,0)
discharge_other or death (%)	3,9 (CI 3,7 - 4,1)	2,7 (CI 2,0 - 3,7)

Outcome characteristics of PNI and control cases measured by GOS (Glasgow Outcome Scale) and frequency of further treatment after primary care discharge. *CI* Confidence Interval of 95%

## Discussion

This retrospective transnational multi-centre study evaluated 49,382 patients with significant upper extremity trauma for concomitant nerve involvement. Our data suggest additional nerve lesions in 3,3% of these patients. Series from various regions all over the world report slightly different PNI prevalence rates which may be caused by heterogeneous socioeconomic and regulatory conditions. 1,12% of unselected trauma patients (*n* = 11.998) presented with PNI in a Mexican trial, whereby - agreeing with our results - upper limb nerves were more commonly involved compared to lower extremity neural structures (7). Other studies from Iran and Canada refer about PNI in 1,3% and 2,8% of multiple trauma patients [4, 6]. Our findings for Central Europe are similar to the results given in the Canadian report possibly due to the comparable background of both economically developed regions. Comprehensive prevalence data of different studies are provided by Table 6. In this study PNI patients were mostly of male gender (78,6%) and generally younger than upper limb trauma victims without nerve injury (mean age 40,6 years versus 47,2 years). These findings are slightly different from the results derived from Castillo-Galvan et al. for Mexican PNI patients with a male fraction of 68% and a distinct lower average age of 27 years [7]. An assessment of severely injured multiple trauma victims (ISS > =16) stated that patients without extremity injuries were on average about five years older than their counterparts with limb involvement [2] which suggests PNI cases comprising an even younger subgroup of an already young trauma population. Several case series of patients with brachial plexopathy, single or combined upper limb nerve trauma support the aforementioned epidemiological observations [11–13]. According to our observations upper extremity trauma with and without PNI can be separated basically by different typical etiological categories. Upper limb PNI resulted from motorcycle and car accidents in the majority of cases (59,4%), whereas main reasons for non-PNI associated upper extremity trauma were falls and car accidents (cumulative 57,6%). Extremities are generally considered to be prone to injury in the context of road traffic accidents [2, 14, 15]. A trial from North India mentioned falls (74,6%) and roadside accidents (14,6%) as most frequently encountered mechanisms for extremity fractures in patients with musculosceletal injuries which matches perfectly the findings for the control group [16]. Corresponding to our results Asplund et al. report on traffic accidents as most important cause of PNI and amputations in Sweden with brachial plexus injury being commonly induced by motorcycle accidents [17], but there are inconsistent findings concerning PNI etiology within the literature. On the one hand traumatic brachial plexopathies were induced by car and motorcycle accidents in 42% of cases in one study [12], but on the other hand there are also series reporting a significant fraction of traumatic nerve lesions being provoked by glass injuries [11] or stab wounds [7] highlighting the importance of the economic and regulatory context of the different study settings. A cut was the main cause of injury in a series of 45 patients suffering from severe hand trauma with about one third of this cohort showing major nerve involvement [18]. Stab wounds, cuts and glass injuries can be categorized as penetrating trauma mechanisms which have also been reported as primary causes for PNI by Saadat et al. [6]. In congruence with the previously mentioned studies our data unveil a considerably higher fraction of penetrating lesions in PNI patients compared to the control group (14,7% versus 3,1%). About one third of our PNI population presented additional head injury which is contradictory to the findings of other series who registered head trauma in 6% and 70% of their patients with brachial plexus injuries, but the results should be interpreted with caution due to the small amount of enrolled patients within these clinical series [12, 19]. Head injuries were even more frequently seen in the control group which could be one possible reason for the observed prolonged intubation time of these patients through associated central respiratory disorder. Furthermore impaired consciousness resulting from head injury may contribute to a certain amount of misclassification with respect to the presence of additional PNI which could explain the higher frequency of craniocerebral trauma in the control group. According to our data upper limb PNI seem to be associated with increased rates of arm fractures compared to the non-PNI control group which is in line with a high rate of ligamentous injuries and fractures affecting the shoulder girdle in a clinical trial concerning brachial plexus injured patients [19]. Complementary a previous registry based study revealed higher rates of PNI in upper compared to lower extremity fractures [6]. Like prior series already stated we also observed a striking coincidence of PNI and vascular lesions which may be caused by the close anatomical proximity of arterial, venous and neural structures in the extremities [11, 13, 19–21]. Despite of equivalent average trauma load in both groups in terms of ISS and mean ICU period we noticed a distinctively increased length of mean hospital stay for PNI compared to control patients which caused considerable additional direct treatment expenditures measured with the cost estimator which has been implemented for the TR-DGU by Lefering et al. [10]. Many studies confirm the high financial burden and resource consumption induced by PNI which seems to be even more significant in upper compared to lower extremity nerve involvement [7, 17, 22]. In case of PNI, expenditures resulting from following lost production may be even more expensive than direct health care costs spent for the initial treatment [18]. With regard to outcome assessment at the point of hospital discharge both groups were mainly classified as GOS 4/5 which means moderate disability (GOS 4) or good recovery (GOS 5), but a significant downgrading from GOS 5 to GOS 4 was ascertained in PNI victims compared to the control cohort which led to increased subsequent need for further inpatient rehabilitation in this patient group. Corroborating this finding many previous studies also report on substantial impairment and diminished quality of life in the long term follow-up after PNI mainly due to reduced functional capacity and permanent primarily neuropathic pain [8, 12, 23, 24]. Hence early detection of accompanying nerve lesions in severely traumatized patients is of crucial importance, because beneficial theapeutic results may be reached through adequate individualized and well-timed microsurgical treatment in many cases [25, 26]. This study may help physicians to suspect PNI in patients coming up with specific trauma mechanisms and injury patterns characterized beforehand which could finally contribute to better functional outcome by early initiation of specialized conservative and surgical care. Besides of the merits of this transnational large-scale survey the main limitation may be its retrospective character. Furthermore we cannot exclude a significant underreporting of PNI as non life-threatening injuries, because the primary focus of the TR-DGU is the evaluation of major trauma in severely injured patients. Moreover traumatized patients who died within the first month after hospital admission were excluded from this study because of possible difficulties regarding meticulous clinical assessment. This cohort is highly suspicious of accompanying PNI due to its extensive trauma load. Hence our presented data concerning PNI incidence rates in upper extremity trauma patients may rather represent minimum values. This aspect is of particular relevance, because most of the previous studies reported on even lower PNI rates presumably due to the same constraints with regard to underestimation.

**Table 6:** PNI prevalence in trauma patients

source	region	period	trauma patients	trauma patients with PNI	PNI prevalence	comment
Noble [4]	Canada	1986–1996	5777	162	2,8%	
Tandon 2007	India	2004–2005	500	3	0,6%	entirely pediatric trauma patients
Taylor [5]	USA	1998	220,593	not available	1,6%	solely upper and lower limb trauma patients
Saadat [6]	Iran	1999–2004	16,753	219	1,3%	
Castillo-Galvan 2014	Mexico	2008–2012	11,998	134	1,1%	
Huckhagel [9]	Central Europe	2002–2015	60,422	1058	1,8%	only lower extremity trauma patients
this study	Central Europe	2002–2015	49,382	1608	3,3%	only upper extremity trauma patients

## Conclusion

This transnational European study reveals a 3,3% rate of concomitant PNI in patients with substantial upper extremity trauma which is slightly higher than expected considering previous American and Far Eastern trials. Patients with additional PNI were generally younger and more likely of male gender compared to their non-PNI counterparts. Upper limb nerve injuries were most frequently caused by motorcycle or car accidents, whereas high or low falls and car crashes induced the main part of upper extremity trauma without PNI. Typical coexisting lesions of PNI patients in this study were humeral or ulnar fractures and vascular lacerations. Despite of equivalent mean trauma severity scores in both cohorts PNI patients showed distinctly extended length of hospital stay combined with further need for inpatient rehabilitation which provokes high financial expenditures for the social welfare system through loss of productivity and healing costs besides the decrease in terms of health related quality of life for the individual patient. Taken together PNI and non-PNI upper extremity trauma can be distinguished by various epidemiological, etiological and outcome characteristics. In the end this study may guide primary care physicians to suspect PNI in patients showing particular trauma mechanisms and patterns expounded beforehand which could finally have an impact on long-term functional performance of trauma victims by accurately timed commencement of specialized conservative and, if applicable, surgical treatment addressing those nerve lesions.

## Abbreviations

AIS: Abbreviated Injury Scale
AUC: Academy for Trauma Surgery
CI: Confidence Interval of 95%
DGU: German Trauma Society
GOS: Glasgow Outcome Scale
ICU/IMC: Intensive Care Unit / Intermediate Care Unit
ISS: Injury Severity Score
NIS: Committee on Emergency Medicine, Intensive Care and Trauma Management of the German Trauma Society
PNI: Peripheral Nerve Injury
SD: Standard Deviation
TR-DGU: TraumaRegister DGU®
